# Reproducibility in Studies on Dental Restorative Materials: A Critical Review

**DOI:** 10.3390/ma19194081

**Published:** 2026-09-24

**Authors:** Matej Par, Zrinka Tarle

**Affiliations:** Department of Endodontics and Restorative Dentistry, University of Zagreb School of Dental Medicine, Gunduliceva 5, 10000 Zagreb, Croatia; tarle@sfzg.unizg.hr

**Keywords:** reproducibility, methodological variability, laboratory testing, dental restorative materials, bond strength, degree of conversion, flexural strength

## Abstract

The evaluation of dental restorative composites relies largely on laboratory studies performed under controlled conditions using standardized testing protocols. However, despite the apparent simplicity and standardization of many commonly used methods, published studies often report surprisingly heterogeneous results. This critical review examines the issue of reproducibility through three representative laboratory tests that differ in their apparent methodological complexity: bond strength, degree of conversion, and flexural strength. Bond strength testing is well recognized for its limited reproducibility due to numerous technique-sensitive procedures and methodological variables. More surprisingly, similar problems exist in degree of conversion measurements, where seemingly minor analytical differences related to spectroscopic technique and data processing can substantially influence the reported values and even alter the relative ranking of materials. The problem becomes particularly striking for flexural strength, a property commonly regarded as one of the simplest and most standardized laboratory measurements. Evidence from published studies and network meta-analyses demonstrates that the reported flexural strength of the same commercial composite may in specific cases vary by more than 100 MPa between studies, with some well-established materials occasionally failing to meet the minimum requirement specified by ISO 4049. These examples illustrate that methodological variability may become sufficiently large to obscure genuine differences between materials, complicate evidence synthesis, and weaken confidence in laboratory findings. By examining three widely used laboratory tests for resin composites, this review aims to raise awareness of the growing but often overlooked problem of limited reproducibility in laboratory studies on dental composites. Such variability may complicate comparisons between materials, interpretation of individual findings, and the development of reliable evidence.

## 1. Introduction

Research on commercial composite materials is driven by the large number of products available at any given time, as well as the frequent introduction of new materials [1,2]. Existing materials are continuously modified, while new “generations”, “successors”, and supposedly improved versions appear on the market every few years [3,4]. In some cases, the turnover is so fast that a material may be discontinued before its properties have been independently investigated to any meaningful extent [5]. The continuous introduction of new materials accompanied by claims of improved performance or completely new functionalities motivates independent evaluations. Although manufacturers partly publish internal data generated during product development, independent studies are still required to complement industry-generated evidence. Unlike investigations performed or sponsored by manufacturers, academic research is (ideally) conducted without the incentive to promote a product, emphasize its advantages, or position it favorably within a competitive market. Independent studies therefore serve as a counterbalance to the claims made by the manufacturers, providing an additional source of information that is less influenced by commercial considerations.

As the intensive research activity produces a vast and rapidly growing amount of literature [6], the abundance of published data presents a practical challenge to the readers [7]. It is nowadays practically impossible to read and critically examine every study published on a given topic, forcing readers to choose which evidence to trust and which sources to consider authoritative. While researchers may be guided by the reputation of the authors or the journal, clinicians and readers less familiar with the research process may non-selectively trust the published literature simply because it has (at least formally) passed peer review and appears in a “scientific” journal. However, publications are not immune to all sorts of shortcomings, including methodological limitations, honest errors, selective reporting, or (hopefully in rare cases) deliberate misconduct [8]. Regardless of the cause, the unavoidable reality is that readers usually form their opinions and conclusions based on a limited subset of the available evidence, because the volume of information far exceeds what any individual can reasonably evaluate and comprehend.

Most clinicians who are not actively involved in research assume that there is at least some level of consensus on the fundamental truths. For example, glass-ionomer cements are considered inherently caries-protective, dental amalgam is envisioned as an outdated and potentially toxic material, and “bioactive” materials are understood as promising future alternatives to the standard “inert” materials. However, a quick literature review is enough to discover that these widely accepted truths are not nearly as settled. Convincing clinical evidence on the anti-caries effect of glass ionomers is still lacking [9,10,11], the impressive list of beneficial effects reported for “bioactive” restorative composites in laboratory studies does not translate into nearly as impressive clinical outcomes [12], while our working definition of “bioactivity” is so flawed and inconsistent that even amalgam can be classified as a bioactive material [13].

While some degree of heterogeneity is normally expected in clinical studies, where patient-related and environmental variables necessarily remain uncontrollable [14], it is much less intuitive that laboratory studies, especially those performed according to standardized protocols under controlled conditions [15,16], often show a surprisingly high lack of agreement. While reproducibility of laboratory studies is commonly assumed because standardized experimental conditions are expected to minimize confounding factors, in practice many parameters measured in vitro show remarkable variability between studies, laboratories, and operators.

This article aims to explore the issue of reproducibility through three widely investigated properties of dental restorative materials: dentin bond strength, degree of conversion, and flexural strength. These properties are interesting to consider because they represent different levels of apparent measurement complexity:

- Dentin bond strength is generally regarded as a highly complex property because it is measured at the interface between three materials (composite/adhesive/dentin) and is influenced by numerous experimental variables [17,18]. Accordingly, reproducibility limitations are not particularly surprising.
- Degree of conversion appears comparatively simpler because it is derived from quantitative spectroscopic measurements that are often perceived as “objective” [19,20]. It is therefore surprising to see that minor methodological variations can exert a disproportionately large influence on the reported values [21].
- Flexural strength of resin composites is commonly regarded as one of the simplest and most standardized laboratory tests [15]. Nevertheless, even this seemingly straightforward measurement can produce remarkably heterogeneous results among studies [22].

By progressing from a property whose reproducibility limitations are intuitive to one where they seem almost paradoxical, these three properties will be used to argue that the perceived simplicity of a measurement does not necessarily mean reproducibility, and to suggest that the reproducibility problem in laboratory studies deserves much more attention than is currently given.

## 2. Bond Strength: Notorious for Low Reproducibility

Bond strength testing has long been known for its poor reproducibility, both at the level of individual replicates within a study (repeatability) and at the level of comparisons among studies (inter-study reproducibility) [23,24]. At the replicate level, bond strength data commonly show substantial variability within the same experimental group [25]. This is partly a consequence of the dependence of the measured values on numerous small variations in specimen preparation, local differences in dentin structure, and the inherently heterogeneous stress distribution during loading [26,27]. The resulting scatter decreases the precision of the measurement, reduces statistical power and makes it more difficult to identify true differences between materials [28]. At the study level, reproducibility is hindered by the practical impossibility of performing all specimen preparation steps in exactly the same way in another laboratory, even when researchers formally follow the same published protocol. Part of specimen preparation relies on what might be described as non-declarative (or “procedural”) knowledge [29], meaning practical skills that cannot be fully transferred through written descriptions. Procedures such as adhesive thinning by air-blowing, dentin grinding, selection of the dentin level used for bonding, sectioning of microtensile specimens, or positioning of specimens within a testing fixture all contain subtle operator-dependent elements that are difficult (if not impossible) to standardize and fully describe in the materials and methods sections [27,30]. Consequently, two laboratories may follow the “same” protocol while still producing measurably different specimens.

Between-study heterogeneity is further compounded by failure mode classification which relies on subjective visual evaluation using different magnifications [31], and statistical treatment introduces another layer of variability, particularly in cases where bond strength values originate from specimens with mixed or cohesive failure modes [32]. In such cases, the recorded value does not necessarily reflect the strength of the adhesive interface alone, but incorporates contributions from the cohesive strengths of dentin and/or composite. One possible solution is to exclude from the statistical analysis specimens that did not fail exclusively in the adhesive mode. However, this introduces an arbitrary decision on where the boundary between adhesive and mixed failures should be drawn. While such selection may be considered necessary to ensure that the measured values truly reflect interfacial strength, it may equally be regarded as an arbitrary exclusion of data [33].

The consequences of the numerous limitations of bond strength testing for measurement reproducibility are well documented [26,27,34]. The same adhesive evaluated in different laboratories, or even within the same laboratory using different test configurations, may yield substantially different bond strength values. Microtensile, macrotensile, microshear, and macroshear tests commonly produce different numerical outcomes, which can even lead to different conclusions regarding the relative performance of different adhesives. This is best illustrated by the fact that when data from multiple studies are synthesized, the ranking of adhesives may change depending on the bond strength test used [34]. If different test setups produce different rankings of the same adhesives, it becomes difficult to determine which values, and ultimately which conclusions, should be trusted. Nevertheless, bond strength testing remains widely used because no better alternative is currently available, and its results are valuable if interpreted with recognition of the test’s numerous limitations.

## 3. Degree of Conversion: Surprisingly Difficult to Measure Reproducibly

While reproducibility problems related to bond strength testing are intuitive, they are not immediately apparent for degree of conversion (DC). In contrast to bond strength, DC appears to be a more “objective” parameter because it is measured using quantitative spectroscopic techniques and expressed as a numerical value representing the percentage of carbon–carbon double bonds consumed during polymerization [35]. Unlike bond strength testing, there is no biological substrate involved, no technique-sensitive specimen preparation, and no subjective classification of failure modes. DC therefore appears to be exactly the type of property for which excellent reproducibility would be expected.

Nevertheless, DC measurements are considerably less straightforward than they appear. The apparent simplicity of DC calculation, based on the intensity change in a single spectral band, conceals a surprisingly large number of methodological variables that can influence the final result [35]. DC is often considered a technique-independent property, implicitly assuming that approximately the same values would be obtained regardless of the analytical methodology used. In practice, however, reported DC values are strongly influenced by the spectroscopic technique (FTIR or Raman), the sampling mode (ATR-FTIR or transmission FTIR), and the subsequent spectral processing [21]. Even when the same spectroscopic technique is used, spectra may be processed using different baseline definitions, deconvolution procedures, and calculation approaches based on either peak heights or peak areas [36].

The simplest and most obvious aspect of spectral processing concerns the use of either peak height or peak area in calculations [37]. Some methodological studies have reported lower DC values when peak-areas are used instead of peak-heights [38], whereas others have reported the opposite effect [39]. Different deconvolution approaches (or lack thereof) introduce additional variability [38], yet their influence has received little systematic investigation and is very rarely reported. The selection of vibrational bands, both reactive and reference, represents another underappreciated source of variability. Although several studies have demonstrated that band selection can influence the obtained DC values [40,41], this issue has received little attention. It has even been suggested that alternative spectral bands may provide more reliable results than the conventionally used band at 1638 cm^−1^ [41].

All of these methodological choices are often treated as minor technical details and are frequently determined by convenience, laboratory tradition, or the availability of established workflows. Also, details of spectral processing are commonly omitted from publications [42,43,44,45,46,47,48], reflecting the widespread assumption that they have little influence on the final DC values. Most studies specify the type of vibrational spectroscopy and sampling mode (e.g., ATR-FTIR, transmission FTIR, or Raman); however, considerably less details are reported on baseline correction procedures, deconvolution methods, selection of reference bands, or the exact mathematical treatment of spectra. With such superficial reporting, two studies may appear methodologically identical while actually employing substantially different analytical approaches. The resulting variability is then attributed to differences between materials, without considering the possibility that a considerable proportion of the observed variation may originate from the methodology itself.

How strongly these underappreciated methodological variables can influence the obtained DC values was demonstrated in a methodological study in which identical composite specimens were analyzed using several commonly employed spectroscopic techniques and calculation approaches [21]. When prepared under identical conditions in the same laboratory, one of the tested composites showed DC values ranging from 60% to 73% using one set of analytical methods and from 56% to 79% using alternative approaches [21]. These differences were obtained without changing the material, curing protocol, or specimen preparation; only the measuring technique (FTIR or Raman) and spectral processing (using band height or area) were changed. In practical terms, differences of this magnitude are more than sufficient to change the outcome of most comparisons among materials. The same composite specimen may be reported as having considerably different polymerization efficiency depending solely on the analytical approach available to, or preferred by, the researcher.

The problem would be less consequential if different analytical approaches merely shifted the reported DC values upward or downward by a similar amount. In that case, although the numerical values would differ, the relative ranking of materials would remain generally unchanged. However, it has been demonstrated that methodological choices can also affect the relative rankings themselves. For example, the relative performance of flowable and sculptable bulk-fill composites can be reversed depending on the selection of spectroscopic technique: flowable materials showed higher DC by FT-Raman, whereas sculptable materials achieved the highest values by ATR-FTIR [21]. This issue is particularly important because the interpretation of comparative studies on restorative materials usually relies on relative rankings instead of exact numerical values. If the ranking changes depending on the spectroscopic method or seemingly minor calculation details, it becomes unclear which ranking should be trusted. Although the underlying problem is essentially the same as in bond strength testing, it is far less recognized in DC measurements. Unlike bond strength testing, where the uncontrolled variability is widely acknowledged as an inherent limitation, the same problem in DC measurements remains hidden behind the supposed “objectivity” of spectroscopic analysis.

The consequences of limited reproducibility and contradictory findings reported across studies become particularly evident when evidence from multiple studies, nominally methodologically similar, but in reality highly heterogeneous, is synthesized in a meta-analysis. Although not fully representative of the entire literature, these analyses provide a systematically selected and methodologically assessed body of evidence. In a recent network meta-analysis on DC of bulk-fill composites, pooled DC data showed substantial heterogeneity and inconsistency between direct and indirect evidence [49]. The variability between studies was so extensive that identifying statistically significant differences between materials became exceedingly difficult despite pooling a large body of evidence. Paradoxically, pooling the data from multiple studies did not strengthen the evidence but instead produced the opposite effect. The accumulated heterogeneity and inconsistency were so extensive that adding more evidence into a network meta-analysis effectively diluted the signal with noise, making genuine material differences more difficult to identify. Rather than strengthening confidence in the comparative performance of multiple materials, the network meta-analysis demonstrated how accumulated methodological variability can obscure genuine material differences [49].

Importantly, all studies included in the aforementioned network meta-analysis [49] were rigorously selected according to predefined eligibility criteria and were considered methodologically sound based on formal risk-of-bias assessment. No obviously flawed studies were included, and therefore the observed heterogeneity in DC data could not simply be dismissed as a consequence of poor study quality. The variability existed despite the inclusion of studies that were all considered methodologically acceptable.

The example of DC is therefore particularly illustrative of the reproducibility issue. The measurement appears objective and robust because it is derived from data acquired by sophisticated instrumentation and calculated using seemingly straightforward mathematics. However, attempts to synthesize data from a larger number of studies reveal that even a property perceived as methodologically rigorous and objectively quantified can be affected by hidden and insufficiently acknowledged methodological variability.

## 4. Flexural Strength: Remarkably Heterogeneous Results for a “Standardized” Test

The examples of bond strength and DC illustrate how reproducibility problems can arise from complex specimen preparation, where they are expected, as well as from methodological variables whose influence is less obvious. Flexural strength testing presents an even more paradoxical situation. It is regarded as one of the simplest and most standardized in vitro tests for resin composites, requiring only the fabrication of a standardized bar-shaped specimen, loading it until fracture, and calculation of flexural strength from the maximum recorded load [50]. It is therefore particularly striking that such a seemingly straightforward test can produce remarkably heterogeneous results between different studies (Figure 1).

The apparent paradox reflects a common misconception that nominal standardization directly translates into reproducibility [51]. Flexural strength testing is generally considered highly standardized, not least because it is incorporated into ISO 4049 [52]. However, nominal adherence to a standardized testing protocol does not necessarily guarantee good reproducibility. While the test appears straightforward, the measured values are highly sensitive to numerous experimental variables that are often insufficiently controlled, incompletely reported, or simply regarded and dismissed as unimportant.

Unlike many chemical and physical properties, flexural strength is inherently sensitive to defects [53]. Fracture typically initiates at the most critical flaw present at the site of load application, so the measured values are determined more by the probabilistic distribution of flaws than by an idealized “average” property of the material [54]. Consequently, even minor differences in specimen preparation may lead to substantial differences in the measured strength. This means that apparently identical specimens prepared using a nominally standardized protocol can produce surprisingly different results.

Operator-related factors are likely the most underestimated source of variability. In some laboratories, specimen preparation and testing may be performed by undergraduate, graduate, or doctoral students rather than dedicated and properly trained technicians. This practice implicitly assumes that flexural strength testing is simple enough to be performed with minimal training. However, operator learning curves almost certainly lead to minor differences in specimen fabrication, including mold filling, removal of excess material, positioning of the curing light, specimen finishing, and other “handling” variables, all of which contribute to variability. Unlike clinical studies, where operator effects are routinely recognized as important factors influencing the measured outcomes [55], laboratory studies pay much less attention to operator-related variability [56]. Operator consistency is rarely evaluated, and even when it is, attention is directed almost exclusively toward clinical procedures [57,58], with its potential influence on laboratory measurements receiving little consideration. Unless the operator preparing the specimens appreciates that flexural strength is inherently probabilistic and governed by the distribution of critical flaws within the specimen, a major source of variability remains overlooked.

Other protocol-related variables contributing to heterogeneity include specimen geometry, curing conditions, and testing environment. Geometric factors are often regarded as inconsequential because small variations in specimen dimensions are routinely “corrected for” by incorporating the dimensions of individual specimens into the flexural strength calculation. Although it is known that geometric effects cannot be completely accounted for by simple mathematical corrections [59], this practice is widespread and implicitly assumes that such corrections fully compensate for geometric differences. Light-curing represents another important but often overlooked source of variability. Curing units are allowed a 10% deviation from their nominal output [60], and differences are known to exist between individual devices of the same model [61]. Bar-shaped specimens for flexural strength testing are longer than the diameter of curing unit tips and always require multiple overlapping irradiations, which makes uniform energy delivery practically impossible. This inevitably produces spatial heterogeneities in polymer structure within individual specimens, resulting in local variations in mechanical properties that become particularly important in a test whose outcome is governed by the distribution of flaws [62]. Additionally, even seemingly minor factors such as testing specimens in a wet or dry state may affect crack propagation and fracture behavior [63]. Batch-to-batch variation may also contribute to between-study variability. Although each of these variables appears to have a minor effect, their cumulative influence can substantially contribute to between-study variability.

While the reproducibility problem is already apparent from variability between individual studies, it becomes especially obvious when a large body of evidence is synthesized. Similar to the previously discussed network meta-analysis on DC, a recent network meta-analysis compared flexural strength among contemporary composites [22]. Although the pooled analysis identified significant differences between some composite classes, most comparisons between individual materials failed to reach statistical significance despite substantial compositional differences that would be expected to influence flexural strength. Also, material rankings varied markedly between studies, with composites expected to perform better not consistently outperforming those expected to perform worse. Consequently, despite pooling all available evidence, establishing a robust overall ranking of materials proved unexpectedly difficult. As in the network meta-analysis on DC [49], only methodologically sound studies meeting (at least nominally) the requirements of ISO 4049 were included in the analysis. This helped minimize the most obvious methodological shortcomings, suggesting that the observed variability could not simply be explained by poor study quality. Nevertheless, the pooled data showed substantial heterogeneity, wide uncertainty intervals, and overall low confidence in the estimated relative differences among materials. As discussed previously for DC [49], including additional studies did not strengthen the evidence. Instead, the accumulated methodological variability diluted the signal with noise, making genuine differences between materials more difficult to distinguish.

A particularly interesting observation concerns the ISO 4049 threshold of 80 MPa commonly regarded as the minimum acceptable flexural strength for load-bearing restorations [64]. As shown in Figure 2, published studies occasionally report values below this threshold even for well-established commercial composites. Given their long history of successful clinical use, such findings are more likely to reflect limited measurement reproducibility than inadequate material performance. This simple observation illustrates how limited the reproducibility of evaluating one of the most fundamental material properties currently is.

From a data analysis perspective, any empirical measurement comprises three components: true material differences, methodological variability, and measurement error. Ideally, the latter two should be sufficiently small that genuine material differences can be reliably detected. However, when uncontrolled variability becomes substantial, it may obscure or even outweigh the differences between materials, reducing statistical power, producing false-negative findings, altering material rankings, and ultimately leading to apparently contradictory conclusions across studies.

The three examples discussed in this review illustrate this principle at different levels. In bond strength testing, methodological variability may contribute more to the final result than the performance of the adhesive itself. More surprisingly, the same problem occurs in measurements perceived as methodologically more straightforward. For DC, different analytical approaches can produce differences exceeding 20 percentage points for the same material, making it difficult to separate true material differences from methodological influences [21]. The magnitude of the problem is even more striking for flexural strength: the range of values reported for a single composite may exceed the differences commonly observed between entirely different material classes [22]. In particularly illustrative cases, the minimum and maximum values reported for the same commercial composite differ by more than 100 MPa, which is greater than the flexural strength reported for some composites (Figure 3). All three examples illustrate the same fundamental principle: as measurement-related variability increases, it becomes progressively more difficult to determine whether observed differences reflect genuine material behavior. This is particularly important when the findings of an individual study are taken for granted, without considering the extent to which unrecognized (and consequently unreported) methodological variability may have influenced the outcome. Given such extensive heterogeneity in laboratory outcomes, the frequently reported poor correlation between laboratory results and clinical performance [65,66] becomes less surprising.

## 5. Reproducibility as a Fundamental Challenge

The three examples discussed in this review demonstrate that limited reproducibility is not restricted to particularly demanding methodology, but represents a fundamental challenge of in vitro studies, including apparently straightforward tests. Nor is this problem confined to dental materials; it is simply the field in which we have sufficient experience to recognize and describe it. Similar concerns have been raised across numerous other disciplines, from psychology [67], where reproducibility issues may be expected, to chemistry [68], where they seem less intuitive. In a survey of 1576 researchers published by Nature, more than 70% reported having failed to reproduce another scientist’s experiments, over 50% had failed to reproduce their own experiments, and about 90% considered science to be facing at least some degree of reproducibility crisis [69]. Various attempts at replication have reached similarly concerning conclusions, demonstrating limited reproducibility across diverse research fields [70,71] including some arguably more consequential than dental materials, such as cancer research [72]. While the causes of poor reproducibility are certainly multifactorial, researchers have identified publication pressure, selective reporting, and incentives that increasingly favor quantity over quality as important contributing factors, a phenomenon politely described as the “misalignment of incentives in academic publishing” [73].

While this review has focused on the possible causes of poor reproducibility in selected tests used in studies on restorative composites, and broader causes have been discussed elsewhere [69,70,71,74,75], identifying the problem is considerably easier than solving it. Many methodological improvements appear self-evident in theory, for example, the seemingly straightforward recommendation to meticulously follow the standard protocols [15,16], but their practical implementation appears considerably more challenging. Indeed, the immense heterogeneity among studies nominally following the same standardized protocols illustrates how challenging the implementation of standardized tests actually is. Furthermore, the “misalignment of incentives” does not encourage time-consuming efforts to improve reproducibility, instead rewarding higher publication output rather than more robust results [76,77,78]. Combined with the increasing popularity and capabilities of large language models (i.e., “artificial intelligence”), which lower the effort and costs required to produce convincing but potentially unreliable articles, these incentives may further threaten the credibility of the published literature [79,80,81]. Nevertheless, the potential concern relates to inappropriate or uncritical use of these tools rather than to the technology itself.

Beyond the academic implications, considering individual primary studies in isolation also has important practical consequences. In university teaching, one frequently encounters students who have read several papers on a particular topic and regard the reported findings as established scientific truths. Another student, having read a different subset of the available literature, may confidently defend an entirely different, or even opposite, position, equally convinced that it is supported by the evidence. Both students are technically correct in the sense that published studies supporting their respective viewpoints do exist. More generally, the sheer volume of published literature makes it inevitable that readers construct their understanding from only a fraction of the available evidence. Depending on which studies they happen to read, different readers may arrive at markedly different conclusions while all believing they are practicing evidence-based reasoning.

There is an interesting concept known as “researcher degrees of freedom”, describing the flexibility available during study design, data analysis, and reporting, which inevitably contribute to between-study heterogeneity [70]. By analogy, we might imagine “reader degrees of freedom” to describe the unavoidable freedom every reader has to construct an opinion from only a limited subset of a vast and heterogeneous body of published evidence. The selectivity may be either unintentional or intentional, but in either case it contributes to confirmation bias [82], a prevalent phenomenon that extends well beyond research and the academic world [83,84]. While an intuitive solution would be for readers to simply consider more studies until they have sufficient evidence on which to base their conclusions, this approach has its own limitations. As illustrated by the two network meta-analyses discussed above [22,49], attempts to synthesize an increasing number of studies may eventually accumulate so much heterogeneity that the additional evidence no longer strengthens the conclusions but instead weakens them. Readers are therefore left with two equally unsatisfactory alternatives: either their conclusions are based on a limited subset of the available literature, or all available evidence is considered (e.g., in a meta-analysis) and the accumulated heterogeneity becomes so substantial that no robust conclusions can be drawn. The former risks unjustified confidence based on incomplete evidence, whereas the latter may undermine confidence altogether because of overwhelming heterogeneity. In such situations, the most honest conclusion may simply be “I neither know nor think I know”, better known in its paraphrased form as “I know that I know nothing” [85]. This does not necessarily mean that reliable conclusions are impossible, but rather indicates that substantial improvements in methodological transparency and reproducibility are needed to increase confidence in the available evidence (Table 1).

Several limitations of this review should be acknowledged. Because the discussion is focused on three selected laboratory tests, similar reproducibility patterns should not be assumed for other laboratory tests or classes of dental restorative materials. Also, in this narrative review the selection of studies and examples was guided by their relevance to the central argument rather than by a systematic search and predefined eligibility criteria. Finally, the quantitative examples are based on data from the presented studies and should therefore be interpreted as illustrative rather than exhaustive.

## 6. Conclusions

The three laboratory tests discussed in this review differ substantially in their apparent methodological complexity, yet all reveal the same underlying problem: limited reproducibility may substantially influence the measured outcomes and the conclusions drawn from them. Particularly noteworthy is that this problem persists even in apparently simple and standardized laboratory methods, suggesting that reproducibility deserves to be regarded as a fundamental challenge of laboratory studies rather than a problem confined to a few technically demanding tests.

Recognizing this limitation is particularly important when interpreting the findings of individual studies, as unrecognized methodological variability may substantially influence the reported results while remaining invisible to the reader. Accordingly, conclusions should be based on the consistency of the wider body of literature rather than on individual findings in isolation.

As offering any solution to the current situation would be overly ambitious and presumptuous, we chose a more modest aim for this article: simply to raise awareness of the growing but often overlooked problem of limited reproducibility in our field. We hope this will serve as the first step towards solving the problem, whatever the solution may eventually be.

## Figures and Tables

**Figure 1:**
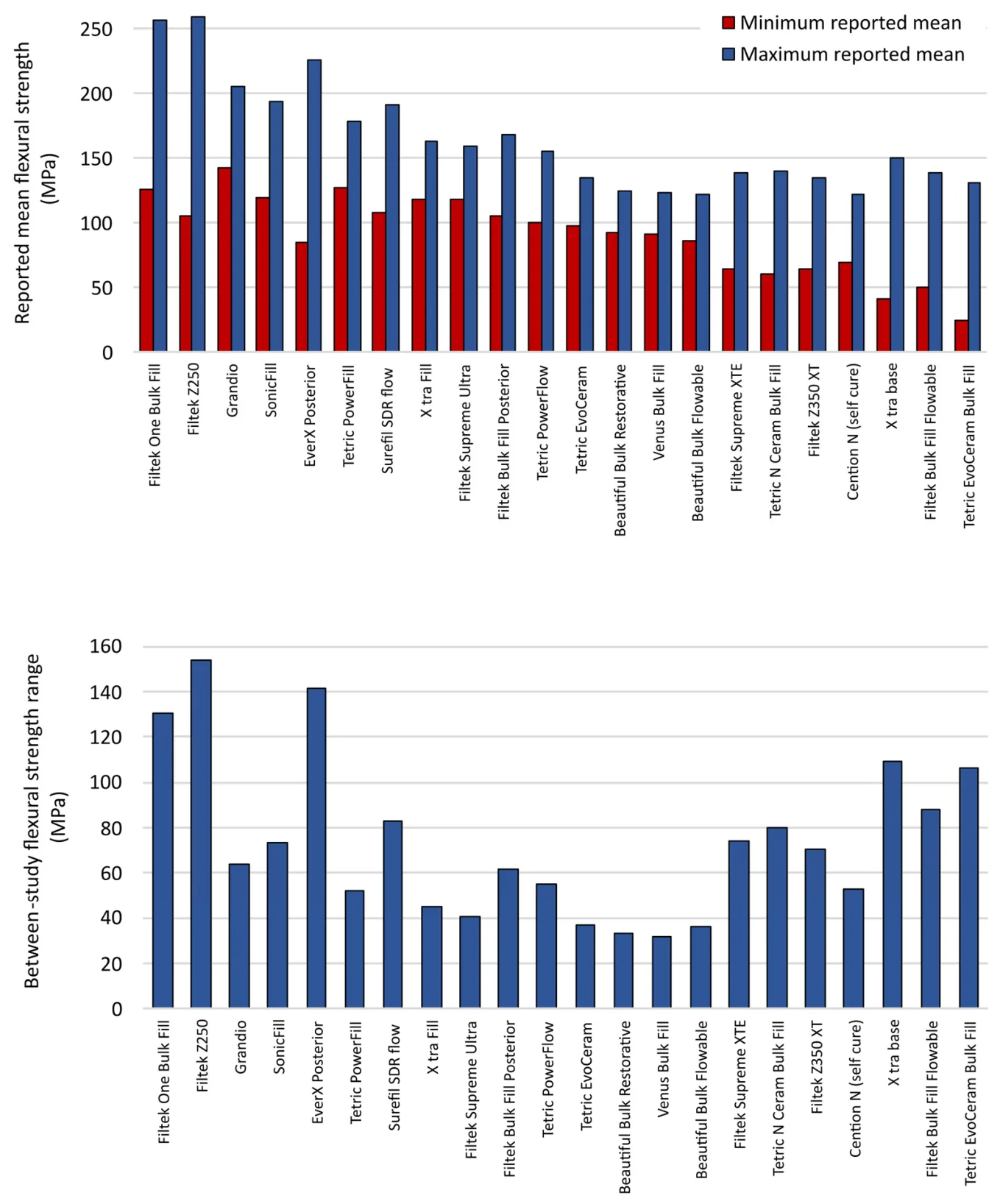
Illustration of the extreme between-study variability in reported flexural strength values. The upper panel presents the minimum and maximum mean values reported for each material, compiled from the primary studies included in the network meta-analysis [22]. The lower panel presents the corresponding ranges (maximum minus minimum mean value). Numbers in parentheses indicate the total number of studies and total number specimens, respectively, for each material: Filtek One Bulk Fill (6, 69), Filtek Z250 (6, 55), Grandio (3, 23), SonicFill (7, 80), EverX Posterior (10, 110), Tetric PowerFill (2, 30), Surefil SDR Flow (18, 204), X-tra Fill (7, 98), Filtek Supreme Ultra (2, 23), Filtek Bulk Fill Posterior (11, 116), Tetric PowerFlow (3, 38), Tetric EvoCeram (3, 43), Beautiful Bulk Restorative (6, 75), Venus Bulk Fill (6, 79), Beautiful Bulk Flowable (5, 61), Filtek Supreme XTE (3, 24), Tetric N-Ceram Bulk Fill (12, 124), Filtek Z350 XT (9, 89), Cention N (self-cure) (3, 35), X-tra Base (7, 91), Filtek Bulk Fill Flowable (14, 151), and Tetric EvoCeram Bulk Fill (12, 148).

**Figure 2:**
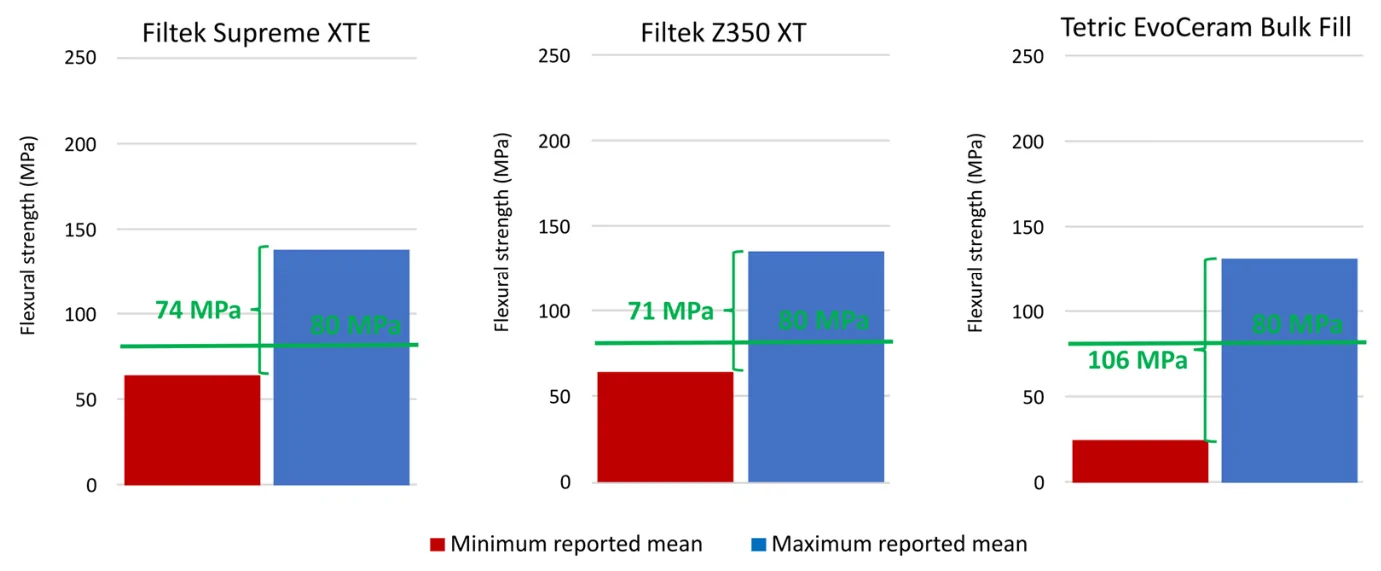
Example of three widely used restorative composites for which some primary studies reported mean flexural strength values below the 80 MPa minimum required by ISO 4049. Minimum and maximum mean values from individual primary studies are shown for each material. The green horizontal line denotes the ISO 4049 threshold, while the bracket indicates the between-study range. The between-study ranges are so large that they exceed the corresponding minimum mean value. Note that the between-study range for Tetric EvoCeram Bulk Fill is even larger than the ISO threshold itself. The figure was prepared using data collected in [22].

**Figure 3:**
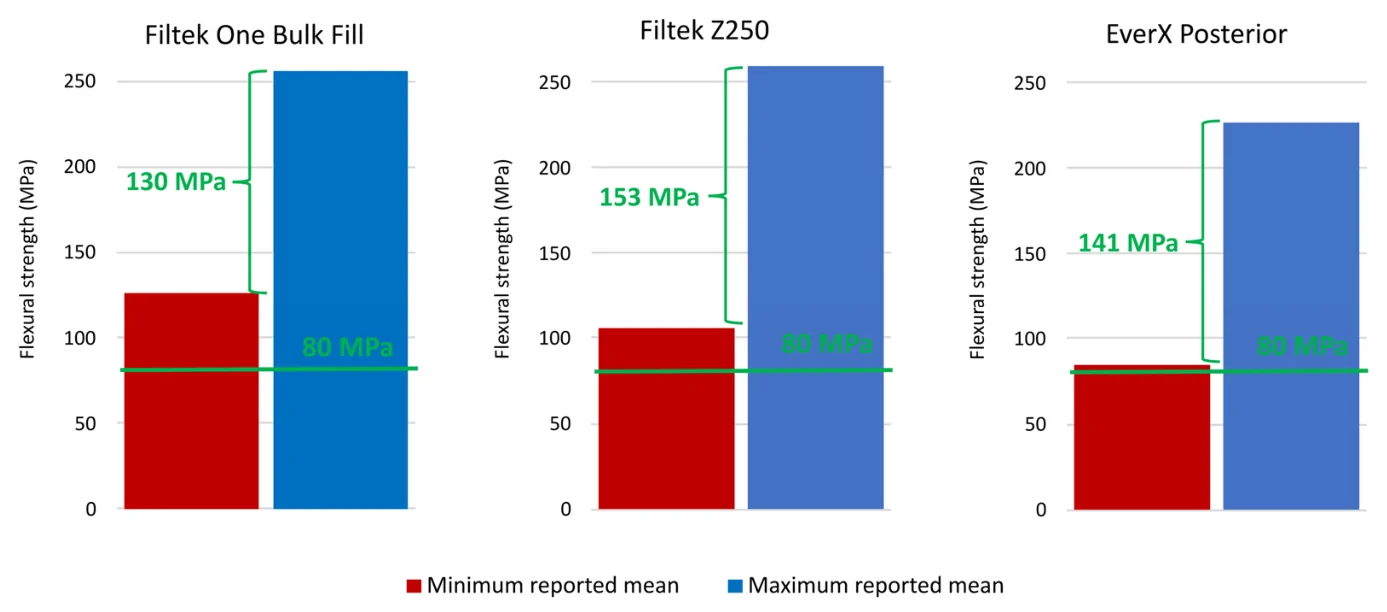
Example of three widely used restorative composites illustrating the extent of between-study variability. Minimum and maximum mean values from individual primary studies are shown for each material. The green horizontal line denotes the ISO 4049 threshold, while the bracket indicates the between-study range. The ranges between the minimum and maximum mean values exceed the flexural strength commonly reported for many commercial composites. For all three materials, the between-study range also exceeds the ISO 4049 threshold of 80 MPa. The figure was prepared using data collected in [22].

**Table 1:** Summary of the main sources of variability identified for dentin bond strength, degree of conversion, and flexural strength, together with potential approaches to improve methodological reporting and standardization.

Laboratory Test	Main Sources of Variability	What Can Be Done to Improve Reporting and Standardization
**Dentin bond strength**	Variability of dentin substrate, tooth source and selection, dentin, tooth age, tooth storage, dentin grinding, adhesive application, solvent evaporation, adhesive thinning, adhesive curing, test configuration (microtensile, macrotensile, microshear, macroshear), specimen geometry, specimen sectioning, positioning within the testing fixture, loading rate, magnification used for failure analysis, treatment of mixed/cohesive failures, potential exclusion of specimens, statistical treatment of failures, statistical treatment of multiple specimens originating from the same tooth, potential pseudoreplication	Detailed description of substrate and specimen preparation, report tooth source and storage, standardize and precisely describe dentin preparation and bonding procedures, report adhesive application and curing procedures, report loading rate and fixture configuration, describe specimen sectioning, clearly define failure-mode classification and magnification used, explicitly state how mixed/cohesive failures and pre-test failures were handled, report data exclusions and their rationale, clearly describe the statistical unit and treatment of multiple specimens from the same tooth, standardize operator training and specimen-preparation procedures
**Degree of conversion**	Spectroscopic technique (e.g., FTIR vs. Raman), sampling mode (e.g., ATR vs. transmission), specimen thickness, deviations from nominal curing unit specifications, timing of measurement after curing, duration of spectral acquisition, signal-to-noise ratio, baseline definition and correction, deconvolution parameters, selection of vibrational bands, peak height vs. peak area, undisclosed laboratory-specific analytical conventions	Precisely report relevant spectral acquisition parameters, report relevant specimen preparation and curing conditions, report timing of measurement after light-curing, describe baseline correction and spectra processing, report deconvolution procedures and parameters, explicitly state whether peak heights or peak areas were used, specify the reactive and reference vibrational bands, use and report a clearly defined and consistent analytical workflow
**Flexural strength**	Mold filling, removal of excess material, specimen finishing and polishing, deviations from nominal specimen geometry, mold characteristics, curing conditions, positioning of curing light, overlapping irradiations, non-uniform energy delivery, specimen storage, testing environment (wet/dry), testing machine and fixture, loading rate, specimen alignment during testing, specimen defects, voids and other fabrication defects	Precisely report fabrication procedures, describe mold filling, excess removal, finishing, and handling, describe the irradiation procedure, including overlapping irradiations, describe specimen orientation and positioning during curing, specify fixture configuration and loading rate, report deviations from nominal specimen dimensions, document specimen defects or exclusion criteria where applicable, maintain consistent operator procedures and training

## Data Availability

No new data were created or analyzed in this study. Data sharing is not applicable to this article.
